# Characteristics of Smart Health Ecosystems That Support Self-care Among People With Heart Failure: Scoping Review

**DOI:** 10.2196/36773

**Published:** 2022-11-02

**Authors:** Rebecca Nourse, Elton Lobo, Jenna McVicar, Finn Kensing, Sheikh Mohammed Shariful Islam, Lars Kayser, Ralph Maddison

**Affiliations:** 1 Institute for Physical Activity and Nutrition, School of Exercise and Nutrition Sciences Deakin University Melbourne Australia; 2 Department of Public Health, Faculty of Health and Medical Sciences University of Copenhagen Copenhagen Denmark; 3 Department of Computer Science, Faculty of Science University of Copenhagen Copenhagen Denmark

**Keywords:** digital health, review, chronic diseases, cardiovascular disease, information technology, digital technology, mobile phone, self-management

## Abstract

**Background:**

The management of heart failure is complex. Innovative solutions are required to support health care providers and people with heart failure with decision-making and self-care behaviors. In recent years, more sophisticated technologies have enabled new health care models, such as smart health ecosystems. Smart health ecosystems use data collection, intelligent data processing, and communication to support the diagnosis, management, and primary and secondary prevention of chronic conditions. Currently, there is little information on the characteristics of smart health ecosystems for people with heart failure.

**Objective:**

We aimed to identify and describe the characteristics of smart health ecosystems that support heart failure self-care.

**Methods:**

We conducted a scoping review using the Joanna Briggs Institute methodology. The MEDLINE, Embase, CINAHL, PsycINFO, IEEE Xplore, and ACM Digital Library databases were searched from January 2008 to September 2021. The search strategy focused on identifying articles describing smart health ecosystems that support heart failure self-care. A total of 2 reviewers screened the articles and extracted relevant data from the included full texts.

**Results:**

After removing duplicates, 1543 articles were screened, and 34 articles representing 13 interventions were included in this review. To support self-care, the interventions used sensors and questionnaires to collect data and used tailoring methods to provide personalized support. The interventions used a total of 34 behavior change techniques, which were facilitated by a combination of 8 features for people with heart failure: automated feedback, monitoring (integrated and manual input), presentation of data, education, reminders, communication with a health care provider, and psychological support. Furthermore, features to support health care providers included data presentation, alarms, alerts, communication tools, remote care plan modification, and health record integration.

**Conclusions:**

This scoping review identified that there are few reports of smart health ecosystems that support heart failure self-care, and those that have been reported do not provide comprehensive support across all domains of self-care. This review describes the technical and behavioral components of the identified interventions, providing information that can be used as a starting point for designing and testing future smart health ecosystems.

## Introduction

Heart failure is associated with a decreased quality of life and increased health care system costs, predominantly because of hospital admissions [1,2]. To prevent deterioration and readmission to hospital, primary and secondary health care providers such as physicians, nurses, and allied health professionals use the practices described in clinical guidelines [3,4]. However, these guidelines are typically long, complex, and subject to changes [5], making them difficult to follow. People with heart failure are also encouraged to practice self-care behaviors to improve their symptoms and manage their health [6,7]. Self-care behaviors include taking medication as prescribed, regular exercise, monitoring symptoms, and titrating medication based on the detection and interpretation of symptoms [6,7]. However, there are numerous barriers to self-care among people with heart failure, including difficulties in recognizing and interpreting symptoms and deciding what course of action to take [8,9].

**Figure 1:**
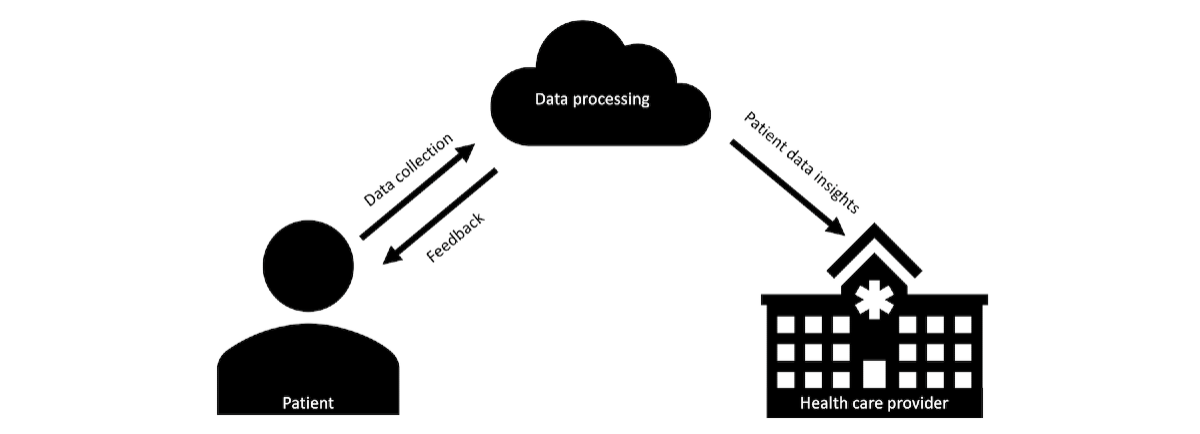
Concept of a smart health ecosystem.

Innovative solutions are required to support health care providers’ decision-making and support people with heart failure to initiate and sustain appropriate self-care behaviors. A recent systematic review of interventions to support self-care among people with heart failure described that effective interventions may have capitalized on interactive telemonitoring devices [10-12], automated and timely responses to participants based on their data [13], and the involvement of health care providers [13,14]. In recent years, improvements in interoperability have driven the integration of more sophisticated technologies (eg, Internet of Things, data storage systems, and artificial intelligence) within health care practice [15,16]. These technologies enable new models of health care that are increasingly being used to assist in the diagnosis, treatment, monitoring and management, including self-care, of people with chronic conditions [17-19]. We refer to this as a smart health ecosystem (Figure 1).

Despite these potential advantages, we do not fully understand the characteristics of smart health ecosystems that support heart failure self-care. In particular, understanding the technical and behavioral components could inform the future design, evaluation, and hypotheses about the mechanisms of action of such interventions. Technical components include the devices used for interaction with the system and data collection and how data are processed and communicated back to people with heart failure and health care professionals. Behavioral components include the active ingredients that change behavior [20]. The behavior change technique taxonomy, version 1 (BCTTv1), provides a list of 93 behavior change techniques (BCTs), which are the smallest components capable of changing behavior [20]. The BCTTv1 can be used to code behavioral components in interventions; for example, setting a target to self-weigh each day would be coded as “goal setting,” receiving information about weekly medication adherence would be coded as “feedback on behavior” and an alarm to remind about taking medication would be coded as “prompts or cues.”

A scoping review can be used to understand a body of literature, identify gaps, and clarify concepts [21]. A preliminary search of MEDLINE, the Cochrane Database of Systematic Reviews, and Joanna Briggs Institute Evidence Synthesis was conducted, and no current or ongoing systematic reviews or scoping reviews on this topic were identified. This scoping review aimed to answer the following questions: (1) What smart health ecosystems to support self-care among people with heart failure are reported in the literature? (2) What self-care behaviors do smart health ecosystems for people with heart failure support? (3) How do smart health ecosystems aim to change or support self-care behaviors?

## Methods

### Study Design

This review was conducted following the Joanna Briggs Institute (JBI) methodology for scoping reviews [22] and adheres to the PRISMA-ScR (Preferred Reporting Items for Systematic Reviews and Meta-Analyses extension for Scoping Reviews) [23]. We did not appraise the methodological quality or risk of bias of the included articles as this is not required for a scoping review.

### Eligibility Criteria

This review was guided by the “population, concept, context” framework suggested by the JBI methodology [22].

#### Population

We considered studies that involved adults (aged ≥18 years) with heart failure living in the community, health care providers (people delivering health care services for people with heart failure), caregivers, and families of people with heart failure, and studies without a population, such as methodological articles, if they addressed the relevant interventions (see concept).

#### Concept and Context

This review considered articles that described, reported the design, or investigated the use of smart health ecosystems (the intervention) that support self-care behaviors in adults (aged ≥18 years) with heart failure living in the community. Although there is no existing definition of such interventions, we considered those with the following elements: (1) data collection using a digital device; (2) automatic processing of data to provide personalized, actionable insights on health and well-being, for example, a recommendation to adjust medication; and (3) health care provider access to data. Interventions that did not explicitly prompt self-care behaviors were excluded, such as those that used an implantable cardiac device or presented data without providing behavioral support or actionable advice. Figure 1 provides a visual representation of this concept.

#### Types of Sources

The following peer-reviewed study designs were considered for this review: experimental and quasi-experimental studies, analytical and descriptive observational studies, and qualitative studies, including intervention design studies. Conference proceedings that reported the listed study designs were considered if they were peer-reviewed, as is the case in many information technology journals. To this end, we excluded conference proceedings that were not peer-reviewed or did not contain a full description of the intervention, such as conference abstracts and posters. Review studies and opinion articles were excluded to limit the studies to technologically feasible interventions.

### Search Strategy

The search strategy was aimed at locating published articles. An initial limited search of MEDLINE and SCOPUS was performed to identify articles on the topic. Text words contained in the titles and abstracts of relevant articles and article index terms were used to develop a complete search strategy for MEDLINE. The search strategy, including all the identified keywords and index terms, was adapted for each included database (Multimedia Appendix 1 contains the search strategies for each database). A research librarian was consulted while developing the search terms and translating the strategy across the databases. The databases searched were MEDLINE (via EBSCO), Embase, CINAHL (via EBSCO), PsycINFO (via EBSCO), IEEE Xplore, and the ACM Digital Library. The searches were conducted in September 2021. The reference lists of included articles were screened for additional papers. For feasibility reasons, only articles published in English were considered. In addition, only articles published between January 2008 and September 2021 (inclusive) were considered. This date range was selected as it accounts for when the Internet of Things was “born” [24].

### Study Selection

Following the searches, all identified articles were collated and uploaded into EndNote X9 (Clarivate Analytics), and duplicates were removed. The citation details of potentially relevant articles were imported into Covidence (Veritas Health Innovation). A total of 2 independent reviewers (RN and JM) screened the titles and abstracts to assess the inclusion criteria. The full texts of the selected articles were assessed in detail against the inclusion criteria by 3 reviewers (RN with EL or JM). During the selection process, disagreements between the reviewers were resolved through discussion or with a third reviewer (EL, JM, or LK).

### Data Extraction

Data from the included articles were extracted by 2 independent reviewers (EL and RN). RN and LK developed the data extraction tool for this review (provided in Multimedia Appendix 2) by adding items relevant to the population, concept and context and research questions to an example form provided by the JBI. Data extracted from all articles included the year of publication, author names, journals, and descriptions of the interventions. For articles that implemented an intervention, details about the participants were extracted. Where multiple articles reported the same intervention, data pertaining to the intervention characteristics were extracted into a single form.

### Data Analysis and Presentation

An inductive content analysis of the intervention descriptions was used to identify and categorize the intervention characteristics. We also deductively coded the intervention descriptions using BCTTv1, a list of 93 techniques categorized into 16 categories [20], to identify the BCTs used in the interventions. RN led the analysis and was supported by EL, LK, and RM, who each had expertise in relevant subject areas (technical, clinical, and behavioral). The results of this review are presented in 2 parts. First, a brief description of the included articles is presented. Next, the characteristics of the interventions are presented.

## Results

### Article Inclusion

**Figure 2:**
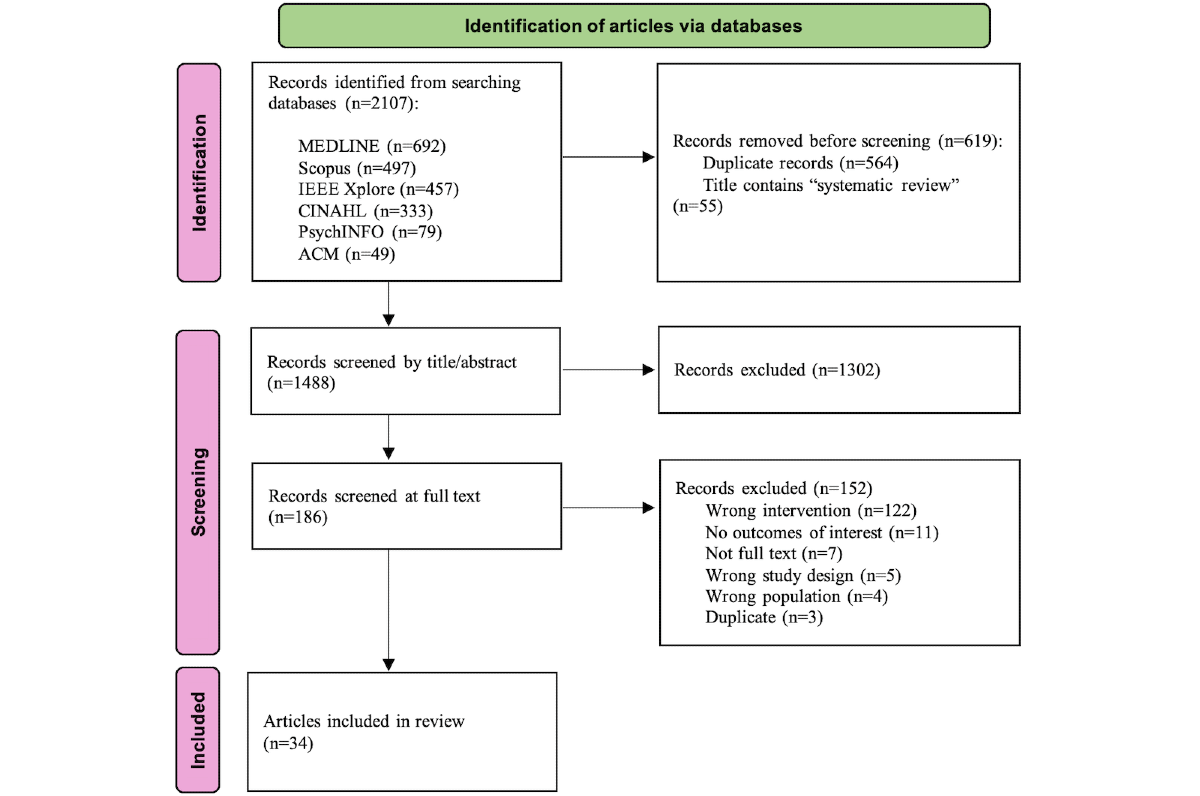
PRISMA-ScR (Preferred Reporting Items for Systematic Reviews and Meta-Analyses extension for Scoping Reviews) flowchart.

A total of 2107 articles were identified from the database searches. After manually removing duplicates (n=564) and using EndNote to remove articles with the words “systematic review” in the title (n=55), 1488 articles remained. The title and abstract screening process left 170 articles for full-text review. A total of 34 articles [13,25-57] representing 13 unique interventions were included in this review. The PRISMA-ScR [58] flowchart in Figure 2 illustrates the selection process. The main reason for excluding articles during full-text review was that they reported an intervention that did not meet our description of a smart health ecosystem.

### Characteristics of the Included Articles

The 34 articles were published between 2009 and 2021, most of which were published during or after 2017 (18/34, 53%). Most of the included articles were published in journals (22/34, 64%), and the remainder were conference proceedings (12/34, 35%). Characteristics of the included articles are provided in Multimedia Appendix 3.

### Intervention Characteristics

#### Overview

As the purpose of this review is to report the characteristics of the 13 included interventions, for the remainder of this review, we will use the metric of the intervention rather than the 34 articles. As such, for interventions reported in multiple articles, only the main article reporting the contents of the intervention (see column 1 in Table 1) is referenced in the subsequent text and tables.

**Table 1:** Intervention mode of delivery.

Intervention name (primary reference)	Mode of delivery for people with heart failure	Mode of delivery for health care provider
CONNECARE [25]	Mobile phone (app)	Web-based platform—accessed by portable tablet
Do Cardiac Health Advanced New Generated Ecosystem (Do CHANGE 2) [27]	Mobile phone (apps, phone call, SMS text messaging), CarePortal, Docobo Ltd.	Web-based portal
HeartCycle Heart Failure Management system [30]	Device connected to television (Philips Motiva)	Web-based platform
HeartMan [35]	Mobile phone (app, phone call), wristband display (custom wristband), pill organizer (PuTwo, 7-Day AM or PM Night Reminder Medi-Planner)	Web application
HeartMapp [40]	Mobile phone (app)	Not reported
Home Automated Telemanagement system [42]	Home unit (notebook computer, PlayStation, Xbox, or Wii)	Clinician unit, email
Medly [48]	Mobile phone (app, automated phone call)	Web dashboard, email
N/A^a^—voice interface technology [52]	Conversational agent (Alexa)	Email and text (alerts)
CardioConsult HF [53]	Health monitor (Turnstall)	SMS text messaging, email, decision support management system (computer)
N/A—a home-based self-management program [54]	Mobile phone (app)	Not reported
N/A—an eHealth self-management intervention [55]	Tablet (app), Respiro, Amiko Digital Health add-on inhaler sensor, face-to-face (individual and group training sessions), phone call	Website
Veta Health [56]	Mobile phone (app)	Veta Health platform (computer)
N/A—an integrated, automatic home-monitoring and assist system [57]	Interactive display wall (video call)	Not reported

^a^N/A: not applicable.

#### Intervention Context

A summary of the contextual characteristics of all 13 interventions is presented in Table 2. Most interventions were designed to address heart failure alone (9/13, 69%) [30,35,40,42,48,52-54,56]. Only 1 intervention was designed for people with both chronic obstructive pulmonary disease and heart failure [55]. In addition, 3 interventions were designed for people with at least one of multiple conditions; people with heart disease (including heart failure) who had received a mechanical circulatory support device [57]; people with coronary artery disease, hypertension, or heart failure [27]; and those with chronic obstructive pulmonary disease or heart failure with a history of hospitalization or who were undergoing major surgery (hip or knee replacement) [25].

Of the 13 interventions, 11 (85%) were tested among participants or involved participants in the intervention development process: 5 in European countries, 4 in the United States, 1 in Canada, and 1 intervention was deployed in a multicenter study in the Netherlands, Spain, and Taiwan (Table 2).

**Table 2:** Characteristics of included interventions.

Name and description (primary reference)	Target condition	Country
CONNECARE—a mobile health–enabled integrated care model [25]	COPD^a^, HF^b^	Spain
Do Cardiac Health Advanced New Generated Ecosystem (Do CHANGE 2)—a personalized digital behavioral intervention program [27]	CAD^c^, HF, HT^d^	Netherlands, Spain, Taiwan
HeartCycle Heart Failure Management System—a personalized disease management care system [30]	HF	N/A^e^
HeartMan—a personal health system [35]	HF	Belgium, Italy
HeartMapp—a theory-based mobile app [40]	HF	United States
Home Automated Telemanagement system—a pervasive telemedicine application [42]	HF	United States
Medly—a mobile phone–based heart failure telemonitoring program [48]	HF	Canada
N/A—voice interface technology [52]	HF	United States
CardioConsult HF—a computerized decision support system [53]	HF	Netherlands
N/A—a home-based self-management program [54]	HF	N/A
N/A—an eHealth self-management intervention [55]	COPD, HF	Netherlands
Veta Health—a hybrid mHealth model [56]	HF	United States
N/A—an integrated, automatic home-monitoring and assist system [57]	Heart disease (including HF) with mechanical circulatory support devices	Germany

^a^COPD: chronic obstructive pulmonary disease.

^b^HF: heart failure.

^c^CAD: coronary artery disease.

^d^HT: hypertension.

^e^N/A: not applicable.

#### Mode of Delivery

Most interventions were delivered entirely digitally (12/13, 92%), and 1 (8%) intervention included a face-to-face component (we did not consider study or trial enrollment sessions), which included individual and group training sessions [55]. Digital modes of delivery included applications or programs available on mobile phones (7/13, 54%) [25,27,35,40,48,54,56], tablets (1/13, 8%) [55], conversational agents (1/13, 8%) [52], notebook computers (1/13, 8%) [42], televisions (1/13, 8%) [30], interactive walls (1/13, 8%) [57], and gaming systems (Microsoft Xbox, Sony PlayStation, and Nintendo Wii; 1/13, 8%) [42]. In addition, existing medical platforms (CarePortal by Docobo, Motiva by Philips, a Tunstall health monitor, and Veta Health) were used in 4 interventions [27,30,53,56], with the Motiva system being adapted by the study group [30]. Furthermore, the interventions used text messages, emails, automated phone calls, and wristband displays as communication tools. More recent interventions used portable devices, such as mobile phones, whereas older interventions used devices placed in the home (eg, gaming systems). Most interventions used a single device as the mode of delivery (10/13, 77%) [25,30,35,42,48,52-54,56,57], whereas 23% (3/13) of interventions [27,35,55] leveraged more than one. Health care providers interacted with the interventions through websites and apps hosted on various devices and received alerts by text messages and emails, but this was less clearly reported in the intervention descriptions.

#### Features for People With Heart Failure

All interventions included 2 features: provision of automated feedback (13/13, 100%) [25,27,30,35,40,42,48,52-57] and monitoring that required manual input (13/13, 100%) [25,27,30,35,40,42,48,52-57]. Additional features were integrated monitoring (11/13, 85%) [25,27,30,35,40,48,53-57], presentation of data (11/13, 85%) [25,27,30,35,40,42,48,54-57], education (10/13, 77%) [25,27,30,35,40,42,53-56], reminders (7/13, 54%) [35,40,48,52,54-56], integrated communication with health care providers (5/13, 38%) [25,42,54,56,57], and psychological support (3/13, 23%) [27,35,55]. None of the interventions delivered all features (range 3-7). Table 3 provides a summary and examples.

**Table 3:** Features for people with heart failure (N=13).

Feature	Value, n (%)	Primary reference for intervention	Examples (not a comprehensive list)
Automated feedback	13 (100)	[25,27,30,35,40,42,48,52-57]	Virtual coach with automated feedback [25]; receive “ToDo” messages based on psychological profile and current functioning [27]; actionable feedback about vital signs measurements to help track progress toward personal goals [30]; warnings if measurements are outside certain ranges [35,57]; automated feedback on walking performance [40]; instant feedback based on action plan zone and measurements [42,52]; automatically generated advice to act (eg, sodium and fluid restriction, contact nurse, monitor blood pressure) [53]; feedback on fluid intake [54]; automated messages with action to take (eg, initiate self-treatment, call case manager) [55]; automated responses to data to promote understanding of self-monitoring data [56]
Monitoring (manual input)	13 (100)	[25,27,30,35,40,42,48,52-57]	Symptom reporting questionnaires [25,27,40,52-56]; health surveys [30]; rating intensity of exercise [35]; disease diary [42]; option to record user-specified data [57]
Monitoring (integrated)	11 (85)	[25,27,30,35,40,48,53-57]	Physiological monitoring with devices (eg, Bluetooth-connected blood pressure monitor, weight scales) [25,27,30,35,40,48,53-57]; take photographs of food (monitored by health care professional) [27]
Presentation of data	11 (85)	[25,27,30,35,40,42,48,54-57]	Overview of data collected by sensors and questionnaires [25,27,30,40,42,48,54-57]; dashboards show the percentage of monthly or weekly activities performed [35]
Education	10 (77)	[25,27,30,35,40,42,53-56]	Health education videos (eg, what is heart failure, symptoms to look out for, physical activity video) [25,30]; guidance on how to take electrocardiogram measurement [27]; educational statements and advice on how to modify the diet to make it healthier [35]; randomly generated questions used to test knowledge (learning by teaching) [40]; interactive questions for disease-specific education [51]; education about heart failure [53]; mini educational game and text-based information [54]; in-person training sessions (group and individual) [55]; view educational content [56]
Reminders	7 (54)	[35,40,48,52,54-56]	Reminders to take measurements (eg, weight, blood pressure) [35,40,48,54]; reminders to answer questionnaire [52]; reminders to take medication [35,40,54]; on sensor audio-visual signs to remind about scheduled medication dose [55]; pop-up notifications for measurements and surveys [56]
Integrated communication with health care provider	5 (38)	[25,42,54,56,57]	Messaging with health care team (including the ability to send images and videos) [25,54]; ability to send messages to health care team (stock messages or can type their own) [42]; direct link to health care provider [56]; direct video link to health care provider [57]
Psychological support	3 (23)	[27,35,55]	Receive “ToDo” messages based on psychological profile and current functioning [27]; cognitive behavioral therapy messages based on psychological profile and games to deal with intrusive thoughts [35]; instruction videos with exercises for relaxation [55]

#### Features for Health Care Providers

Health care providers involved in the interventions were case managers, nurses, specialists, nutritionists, psychologists, and general practitioners. Features for these health care providers included support for decision-making and prioritization through providing visualization of information and data that had been collected using sensors and questionnaires (13/13, 100%) [25,27,30,35,40,42,48,52-57], alerts and alarms (eg, for measurements that fell out of range or symptom deterioration (9/13, 69%) [25,30,42,48,52-54,56,57], and by facilitating remote treatment plan changes (5/13, 38%) [25,30,35,42,53]. Although only 38% (5/13) of interventions facilitated in-system communication with people with heart failure (eg, through in-app messaging or a video consultation) [25,42,54,56,57], many intervention descriptions inferred that health care providers would provide direct contact if required. Only one intervention alerted health care providers to any technical problems—a low battery on a weight scale [53].

#### Data Collection

Data collection fell under 4 categories: physiological, symptom, behavioral, and others (Table 4). Only 1 intervention did not collect any physiological data [52], 3 did not collect any information about symptoms [35,54,57], and 3 did not collect data on behaviors [30,48,53]. Data on physiological parameters were collected using commercially available devices. Although most interventions were intended to supply the devices required to collect relevant data, others used devices owned or supplied by people with heart failure [40,48,54,56]. Overall, the content of questionnaires was not clearly reported in the intervention descriptions. Where reported, symptoms included shortness of breath, edema, chest pain, fatigue, palpitations, dizziness, medication side effects, fainting, implantable cardiac device activation, nighttime breathing, and cough. Questionnaires included rating symptoms from absent to severe [30], comparing symptoms to “usual” symptoms [55], and simply reporting the absence or presence of a symptom [25,27,40,42,48,52,53,56]. A conversational agent was used to ask a series of questions that required a yes or no response by 1 intervention [52]; this questionnaire was based on 3 literature sources [59-61]. Although physiological data collection relied on sensors and symptom data on self-reports, behavioral data were collected by both sensors and self-reports. Behaviors monitored by the interventions included physical activity, medication adherence and techniques, sleep, adherence to self-weighing, fluid intake, food consumption, and cooking behavior. Some devices were used to collect more than one parameter; for example, a Fitbit could collect both heart rate and sleep data. Custom-built devices were used in 3 interventions; these devices included a wristband with a photoplethysmography sensor, triaxial accelerometer, and a temperature sensor [35]; a shirt to measure vitals during exercise [30]; a smart spatula to measure cooking behavior and salinity of food being cooked; and a fluid monitor that could be attached to a glass or bottle to gauge the amount of fluid contained [27]. Other data collected were mostly used to further personalize interventions (see the section Tailoring and Personalization). Questionnaires were used to determine personality profiles, comprehension and motivation, depression, and anxiety scores. These devices were used to collect GPS location data, voice recordings, and environmental and humidity data.

**Table 4:** Physiological and behavioral data collection: parameters and measurement tools (N=13).

Parameter			Value, n (%)		Measurement tools in each intervention (primary reference)
**Physiological**					
	Weight	11 (85)		Weight scale, Withings (unspecified model) [25]; Aura 807 scale, Seca [27]^a^; Silje BE 1303 [35]; Self-owned scale [40,48,54,56]^a^; 321P, Lifesource [42]^a^; Bluetooth-enabled weight scales [48,55]; Weight scale, A&D instruments (unspecified model) [53]; Weight scale, Kern (placed under a floor tile) [57]; Network of piezoelectric sensors under floor tiles [57]	
	Blood pressure	8 (61)		Monitor, Withings (unspecified model) [25]; UA-737 Plus, A&D Medical [27]^a^; UA-611, A&D Medical [35]; Bluetooth-enabled blood pressure cuff [48,56]; Monitor, A&D instruments (unspecified model) [53]; Boso sensor integrated into furniture [57]; Unspecified [54]^a^	
	Heart rate	7 (54)		Fitbit Alta HR, Fitbit [27]; Wristband sensor, BITTIUM, Oulo (custom developed for study) [35]; BioHarness-3 chest strap [40]; Boso sensor integrated into furniture [57]; Unspecified [48,54,56]^a^	
	Temperature	3 (23)		Monitor, Withings (unspecified model) [25]; wristband sensor, BITTIUM, Oulo (custom developed for study) [35]; High precision infrared camera, Flir Systems (placed on wall) [57]	
	Blood oxygen saturation	2 (15)		Monitor, Withings (unspecified model) [25]; Bluetooth-enabled pulse oximeter [56]	
	Heart rate variability	1 (8)		Wristband sensor, BITTIUM, Oulo (custom developed for study) [35]; BioHarness-3 chest strap [40]	
	Electrocardiogram	1 (8)		CarePortal, Docobo [27]	
	Heart rate (sleep)	1 (8)		Beddit 3 [27]	
	Breathing rate (sleep)	1 (8)		Beddit 3 [27]	
	Galvanic skin response	1 (8)		Wristband sensor, BITTIUM, Oulo (custom developed for study) [35]	
	Coagulation	1 (8)		CoaguChek, Roche Diagnostics integrated into furniture [57]	
	Unspecified	1 (8)		Unspecified devices to measure vital parameters [30]	
**Behavioral**					
	Physical activity (eg, step count, accelerometry)	5 (38)		Fitbit Alta HR, Fitbit [27]; Fitbit (unspecified model) [25,55]; Wristband sensor, BITTIUM, Oulo (custom developed for study) [35]; BioHarness-3 chest strap [40]	
	Medication adherence	4 (31)		Question on number of pills remaining, adherence calculated based on deviation from expected number [35]; voice response questionnaire [52]; Respiro, Amiko Digital Health (add-on sensor for inhaler) [55]; unspecified questionnaire [56]	
	Salt intake	2 (15)		CooKiT, study developed device (sodium and potassium sensor) [27]; voice response questionnaire [52]	
	Fluid intake	2 (15)		FLUiT study developed device [27]; self-report intake [54]	
	Medication technique	1 (8)		Respiro, Amiko Digital Health (add-on sensor for inhaler) [55]	
	Eating behavior	1 (8)		Take photographs of food 3 times a day in mobile app [27]	
	Self-weighing	1 (8)		Voice response questionnaire [52]	
	Cooking behavior	1 (8)		CooKiT, study developed device (motion sensor spatula) [27]	
	Sleep	1 (8)		Beddit 3 [27]	
	Adherence (unspecified)	1 (8)		Questionnaire [42]	

^a^Denotes manual input required.

#### Tailoring and Personalization

Tailoring and personalization were driven by human input or by algorithms and machine learning techniques (Table 5 provides a summary and examples). All interventions provided tailored advice based on the data collected. Interventions leveraged multiple processing techniques such as rule-based reasoning, machine learning, and comparing data to parameters set by clinical guidelines, historical trends, or expert data from health care providers (3/13, 23%) [42,48,57]. In addition, 10 interventions [25,27,30,35,40,42,48,52,54,55,57] demonstrated enhanced personalization, including tailoring intervention content (5/13, 38%) [25,35,40,54,62], timing of delivery (3/13, 23%) [27,35,52], monitoring devices (3/13, 23%) [25,27,55], and the mode of delivery (1/13, 8%) [27].

**Table 5:** Tailoring and personalization (N=13).

Features	Value, n (%)	Primary reference for intervention	Examples (not a comprehensive list)
Advice	13 (100)	[25,27,30,35,40,42,48,52-57]	Advice based on risk stratification (calculated by assessing personal characteristics and environment) [25]; messages personalized based on personality profile, social opportunity, variety and activity, and physical activity status [27]; predictive models recommended actions related to temperature and humidity [35]; built-in algorithm analyzed weight and symptom data and gave feedback depending on status [40]; in case of deviation from predefined values, system asked about symptoms and then provides advice based on heart failure guidelines [53]
Intervention content	5 (38)	[25,30,35,40,54]	Cycloergometry or 6-minute walk test used to assess fitness, appropriate exercises given based on test results [35]; questions on current lifestyle and behavior determined which education topics are presented [30]
Alert parameters	3 (23)	[42,48,57]	Adaptive feature extraction—can be updated with current user or expert data [57]
Timing of delivery	3 (23)	[27,35,52]	Physical activity recognition from accelerometer in wristband allowed for psychological interventions to be delivered at an appropriate moment [35]; reminder alarm time could be scheduled at a preferred time [52]
Monitoring devices	3 (23)	[25,27,55]	Devices determined by health care team [25,27,55]
Mode of delivery	1 (8)	[27]	Options for mode of delivery of messages [27]

#### Theoretical Grounding

Of the 13 interventions, 7 (54%) were developed with guidance from one or more theories: self-regulation theory [30], cognitive behavioral therapy [35], theory of cognitive dissonance [35], Do Something Different behavior change program [27], the multidimensional framework of patient engagement [40], intervention motivation-behavior model [40], chronic disease care model [42], the framework for Self-Care in Chronic Illness [48], activity theory [54], and multiple theories used to promote engagement with educational content [40]. The details of the theories corresponding to each intervention are available in Multimedia Appendix 4. Finally, 4 interventions included educational content or advice based on clinical guidelines and recommendations [30,40,53,54].

#### Behavior Change Techniques

A total of 34 unique BCTs from BCTTv1 were identified in the 13 interventions, with an average of 12 BCTs per intervention (range 7-26). Table 6 provides a summary of the BCTs and their corresponding categories from the BCTTv1 that we identified for each intervention. A total of 8 BCTs were identified in at least 75% of the interventions: adding objects to the environment (13/13, 100%), self-monitoring of outcome(s) of behavior (12/13, 92%), biofeedback (12/13, 92%), pharmacological support (12/13, 92%), feedback on behavior (11/13, 85%), prompts and cues (11/13, 85%), self-monitoring of behavior (10/13, 77%), and social support (10/13, 77%).

**Table 6:** Summary of behavior change techniques used in the interventions according to behavior change technique taxonomy, version 1 (BCTTv1) (N=13).

Behavior change technique (numbering according to BCTTv1)		Value, n (%)	Primary reference for intervention
**1. Goals and planning**			
	1.1. Goal setting (behavior)	4 (31)	[25,30,35,54]
	1.2. Problem solving	2 (15)	[35,55]
	1.4. Action planning	7 (54)	[30,35,42,48,53,55,56]
	1.5. Review behavior goal(s)	1 (8)	[35]
	1.6. Discrepancy between current behavior and goal	5 (38)	[25,35,54-56]
**2. Feedback and monitoring**			
	2.1 Monitoring of behavior without feedback	1 (8)	[27]
	2.2. Feedback on behavior	11 (85)	[25,27,30,35,40,42,52,54-57]
	2.3. Self-monitoring of behavior	10 (77)	[25,30,35,40,42,52,54-57]
	2.4. Self-monitoring of outcome(s) of behavior	12 (92)	[27,30,35,40,42,48,52-57]
	2.6. Biofeedback	12 (92)	[25,27,30,35,40,42,48,53-57]
	2.7. Feedback on outcome(s) of behavior	9 (69)	[25,27,30,40,42,48,52,53,57]
**3. Social support**			
	3.1. Social support (unspecified)	10 (77)	[25,27,30,35,40,52,54-57]
**4. Shaping knowledge**			
	4.1. Instruction on how to perform the behavior	5 (38)	[25,27,35,42,55]
**5. Natural consequences**			
	5.1. Information about health consequences	3 (23)	[35,40,55]
	5.4. Monitoring of emotional consequences	1 (8)	[35]
	5.5. Anticipated regret	1 (8)	[35]
	5.6. Information about emotional consequences	1 (8)	[40]
**6. Comparison of behavior**			
	6.1. Demonstration of the behavior	2 (15)	[25,55]
**7. Associations**			
	7.1. Prompts or cues	11 (85)	[27,30,35,40,48,52-57]
**8. Repetition and substitution**			
	8.1. Behavioral practice or rehearsal	3 (23)	[35,54,55]
	8.2. Behavior substitution	2 (15)	[27,35]
	8.3. Habit formation	2 (15)	[35,54]
	8.4. Habit reversal	2 (15)	[27,35]
	8.7. Graded tasks	2 (15)	[30,35]
**9. Comparison of outcomes**			
	9.1. Credible source	5 (38)	[25,40,42,54,55]
**10. Reward and threat**			
	10.2. Material reward (behavior)	1 (8)	[54]
	10.3. Nonspecific reward	1 (8)	[25]
	10.4. Social reward	4 (31)	[30,35,54,56]
**11. Regulation**			
	11.1. Pharmacological support	12 (92)	[25,30,35,40,42,48,52-57]
	11.2. Reduce negative emotions	3 (23)	[35,40,55]
**12. Antecedents**			
	12.1. Restructuring the physical environment	1 (8)	[35]
	12.3. Avoidance or reducing exposure to cues for the behavior	2 (15)	[35,55]
	12.4. Distraction	1 (8)	[35]
	12.5. Adding objects to the environment	13 (100)	[25,27,30,35,40,42,48,52-57]

## Discussion

### Principal Findings

This scoping review aimed to understand the extent of the literature on, and the characteristics of, smart health ecosystems that support self-care behaviors among people with heart failure. We identified 34 articles describing 13 interventions. Most of the articles were published during or since 2017. Only 61% (8/13) of interventions in this review had undergone effectiveness testing or implementation at the point of the search, highlighting the novelty of this research area. We expect that the literature published in this area will increase as technologies are developed, tested, and integrated into health care delivery.

Heart failure self-care requires a person to recognize their symptoms [7]. Several devices and questionnaires were used to monitor signs and symptoms but still required a degree of manual input. As these interventions require daily use, future designs may consider using more sophisticated data processing techniques to reduce the workload of people with heart failure. For example, 1 intervention used machine learning techniques to infer physiological and psychological status, which potentially reduced the need to use monitoring tools multiple times a day [35]. With more advanced data collection and processing, privacy and security issues may concern stakeholders. Hence, as with any intervention embedded in a health care system, rigorous data management and storage protocols must be implemented.

We found that interventions leveraged commercially available or hidden devices (embedded within furniture [57]) which may reduce condition-related stigmatization and a feeling of disease being in the home compared with medical devices [63-65]. However, devices that are not portable could lead people with heart failure to feel as though they are confined to the home, or a spot within the home, because the device cannot travel with them. Some interventions have used portable devices that will allow for mobility. Commercially available devices may have limited validity in people with chronic conditions. For instance, Fitbits were used to track steps; however, a study testing the use of Fitbits to measure steps in free-living conditions concluded that although clinicians may use the data to motivate people with heart failure to walk more, the device did not meet a threshold for validity [66]. This may present a safety concern if automated advice is based on invalid data, especially without review by a health care provider. A recently developed framework for choosing devices for mHealth interventions might provide a starting point for future intervention designs [67]. Moreover, despite more people developing competence in interacting with digital technology, there are still groups of people who are not confident, have poor digital literacy or do not have access to the internet. Smart health ecosystems risk exacerbating health inequalities without careful consideration by intervention developers and policy makers [68,69].

In addition to monitoring, many interventions included features that may aid people with heart failure in recognizing and interpreting their symptoms. These features included the provision of education and coaching; for example, by providing videos demonstrating what a particular symptom looks like before filling out a symptom questionnaire. Finally, by providing personalized automated feedback, interventions may help people with heart failure to take evidence-based actions to promote health and prevent further deterioration.

Compared with clinical guidelines [3,4] and a list of practical self-care behaviors developed by the European Society of Cardiology [6], the interventions reported in this review covered a broad range of self-care behaviors. However, no single intervention has provided comprehensive support across all recommendations. As self-care can be practiced in both healthy and ill states [7], there is an opportunity for future interventions to support people before their symptoms deteriorate by providing features that promote health maintenance and adherence. The interventions in this review included BCTs that fall under the categories of “goals and planning,” “feedback and monitoring,” and “antecedents.” A study analyzing digital health behavior change technologies from 2000 to 2018 also reported that the most common BCTs identified in such interventions were related to goal setting and self-monitoring [70]. However, a study that identified BCTs to overcome barriers to self-care among people with heart failure included those in the categories of “social support,” “shaping knowledge,” “natural consequences,” and “repetition and substitution” [9]. The adaptability and flexibility of smart health ecosystems can allow for innovative functions and features, including the delivery of additional BCTs.

The articles reported limited information on how the interventions supported the health care providers. From the evidence provided, interventions presented health care providers with clear and timely information about health status, prompting clinical intervention when required. The interventions were designed to identify early signs of deterioration and to enhance existing services rather than replace them. One limitation to using automated decision support in health care is automation bias and complacency, where health care providers rely on the technology and do not perform as diligently as they would without it [71]. Future interventions should consider ways to avoid this potential problem. Nevertheless, we hypothesize that well-designed interventions may streamline health care providers’ work as the number of people with heart failure increases. In addition, by providing automated advice to people with heart failure, less frequent support from health care providers may be required. The normalization process theory framework [72] may inform the design and evaluation of future interventions to understand and enhance how they are integrated into users’ daily habits and routines [73-75]. Finally, to prevent siloed care, interventions should combine data with electronic health records and facilitate communication with other members of the care team.

### Implications for Research

Gaps in the literature related to smart health ecosystems for people with heart failure were identified. Few interventions provided comprehensive self-care support across all self-care behaviors or considered the presence of comorbidities that may interact with signs, symptoms, and self-care behavior among people with heart failure. A recent review of self-care interventions for chronic conditions also reported this finding [76]. Future interventions should incorporate support for a wide range of behaviors that can be tailored to individual needs. Technologies and data analyses are now advanced enough to consider the interaction of comorbidities with heart failure, and as the number of people with more than one condition increases, interventions could target people with multiple conditions. Moreover, most studies were conducted in the United States and Europe. Research should be conducted in additional regions of the world and, thus, different health care settings to provide deeper insights. Further research should include a systematic review to investigate the effects of smart health ecosystems on people with heart failure.

### Strengths and Limitations

To our knowledge, this scoping review is the first to examine the characteristics of smart health ecosystems to support self-care in people with heart failure. We conducted an extensive literature search using 5 health science and information technology databases and considered a broad range of study designs. On the basis of the number of published articles identified in our original search, we chose not to extend the search to include gray literature or patent databases; however, this may have uncovered upcoming, promising interventions. Searching the literature for “smart health ecosystems” was difficult because of the diversity in the language used to describe such interventions. Consequently, some articles may have been missed. Two reviewers extracted data from the included articles and coded the intervention characteristics, but only one reviewer coded the intervention descriptions against BCTTv1. In this instance, coding was kept close to the manifest meaning of the text, and other reviewers with expertise in this area were consulted throughout the process. Finally, our analysis was based on information in the articles and their published protocols, but we may have missed intervention characteristics due to unclear descriptions.

### Conclusions

This scoping review identified and described the characteristics of 13 smart health ecosystems that support self-care among people with heart failure. We have outlined the behavioral and technical components of the interventions and have highlighted gaps in the provision of support and the literature. We discuss opportunities to augment smart health ecosystems and suggest further research to assess their effectiveness. Alongside other literature, this information can be used to assist in the development and evaluation of future interventions.

## Appendix

Multimedia Appendix 1 Search strategies for the MEDLINE, Embase, CINAHL, PsycINFO, IEEE Xplore, and ACM Digital Library databases.

Multimedia Appendix 2 Data extraction form.

Multimedia Appendix 3 Summary characteristics of included articles.

Multimedia Appendix 4 Intervention characteristics.

## Abbreviations

BCT: behavior change technique
BCTTv1: behavior change technique taxonomy, version 1
JBI: Joanna Briggs Institute
PRISMA-ScR: Preferred Reporting Items for Systematic Reviews and Meta-Analyses extension for Scoping Reviews
