# Supplementary material for: Characteristics of Smart Health Ecosystems That Support Self-care Among People With Heart Failure: Scoping Review
Source: JMIR Cardio. 2022 Nov 2;6(2):e36773. doi: 10.2196/36773 (PMC9669885; doi:10.2196/36773)
Supplement: Multimedia Appendix 1 [file cardio_v6i2e36773_app1.docx]

#### Multimedia Appendix 1

#### Search strategies for MEDLINE, Embase, CINAHL, PsycINFO, IEEE Xplore, and ACM Digital Library databases

**Database 1: MEDLINE (n=692)**

| **Search no.** | **Construct** | **Search terms** |
| --- | --- | --- |
| 1 | Health | ( TI health OR AB health ) OR ( TI healthcare OR AB healthcare ) |
| 2 | System | ( TI deliver* OR AB deliver* ) OR ( TI model* OR AB model* ) OR ( TI ecosystem* OR AB ecosystem* ) OR ( TI network* OR AB network* ) OR ( TI system* OR AB system* ) OR ( TI management OR AB management ) OR ( TI home OR AB home ) OR ( TI integrated OR AB integrated ) OR ( TI service* OR AB service* ) OR ( TI remote OR AB remote ) |
| 3 | MeSH | (MH "Delivery of Health Care") OR (MH "Telemedicine") OR (MH "Telerehabilitation") OR (MH "Remote Consultation") OR (MH "Delivery of Health Care, Integrated") |
| 4 |  | 1 AND 2 |
| 5 |  | 3 OR 4 |
| 6 | Technology | ( TI device* OR AB device* ) OR ( TI "tele medicine" OR AB “tele medicine" OR TI telemedicine OR AB telemedicine ) OR ( TI "e health" OR AB “e health" OR TI ehealth OR AB ehealth ) OR ( TI "m health" OR AB “m health" OR TI mhealth OR AB mhealth ) OR ( TI mobile OR AB mobile ) OR ( TI "internet of things" OR AB “internet of things" ) OR ( TI "IoT" OR AB "IoT" ) OR ( TI technolog* OR AB technolog* ) OR ( TI smart OR AB smart ) OR ( TI ubiquitous OR AB ubiquitous ) OR ( TI digital OR AB digital ) |
| 7 | MeSH | (MH "Internet") OR (MH "Internet-Based Intervention") OR (MH "Internet of Things") OR (MH "Internet of Things") OR (MH "Artificial Intelligence+") OR ("Digital Technology") |
| 8 |  | 6 OR 7 |
| 9 |  | 5 AND 8 |
| 10 |  | ( TI "self care" OR AB “self care" OR TI "selfcare" OR AB “selfcare" ) OR ( TI monitor* OR AB monitor* ) OR ( TI "self management" OR AB “self management" ) OR ( TI maintenance OR AB maintenance ) OR ( TI maintain OR AB maintain ) OR ( TI behavior OR AB behavior ) OR ( TI behavior OR AB behavior ) |
| 11 |  | (MH "Health Behavior") OR (MH "Treatment Adherence and Compliance") OR (MH "Self-Management") OR (MH "Self Care") OR (MH "Monitoring, Physiologic") |
| 12 |  | 10 OR 11 |
| 13 |  | 9 AND 12 |
| 14 |  | TI ( myocardium OR myocardial OR ventric* OR cardial OR heart OR cardio* ) OR AB ( myocardium OR myocardial OR ventric* OR cardial OR heart OR cardio* ) |
| 15 |  | TI ( failure OR decompensat* OR insufficien* OR dysfunction* ) OR AB ( failure OR decompensat* OR insufficien* OR dysfunction* ) |
| 16 |  | (14 N2 15) OR (MH "Heart Failure") |
| 17 |  | 13 AND 16 |
| 18 |  | Restrict to English & published since 2008 |

**Database 2: Cinahl (n=333)**

| **Search no.** | **Construct** | **Search terms** |
| --- | --- | --- |
| 1 | Health | ( TI health OR AB health ) OR ( TI healthcare OR AB healthcare ) |
| 2 | System | ( TI deliver* OR AB deliver* ) OR ( TI model* OR AB model* ) OR ( TI ecosystem* OR AB ecosystem* ) OR ( TI network* OR AB network* ) OR ( TI system* OR AB system* ) OR ( TI management OR AB management ) OR ( TI home OR AB home ) OR ( TI integrated OR AB integrated ) OR ( TI service* OR AB service* ) OR ( TI remote OR AB remote ) |
| 3 | CINAHL heading | (MH "Delivery of Health Care") OR (MH "Telemedicine") OR (MH "Telerehabilitation") OR (MH "Remote Consultation") OR (MH "Delivery of Health Care, Integrated") OR (MH "Home Health Care Information Systems") |
| 4 |  | 1 AND 2 |
| 5 |  | 3 OR 4 |
| 6 | Technology | ( TI device* OR AB device* ) OR ( TI "tele medicine" OR AB “tele medicine" OR TI telemedicine OR AB telemedicine ) OR ( TI "e health" OR AB “e health" OR TI ehealth OR AB ehealth ) OR ( TI "m health" OR AB “m health" OR TI mhealth OR AB mhealth ) OR ( TI mobile OR AB mobile ) OR ( TI "internet of things" OR AB “internet of things" ) OR ( TI "IoT" OR AB "IoT" ) OR ( TI technolog* OR AB technolog* ) OR ( TI smart OR AB smart ) OR ( TI ubiquitous OR AB ubiquitous ) OR ( TI digital OR AB digital ) |
| 7 | CINAHL heading | (MH "Internet") OR (MH "Internet-Based Intervention") OR (MH "Internet of Things") OR (MH "Internet of Things") OR (MH "Artificial Intelligence+") OR ("Digital Technology") |
| 8 |  | 6 OR 7 |
| 9 |  | 5 AND 8 |
| 10 |  | ( TI "self care" OR AB “self care" OR TI "selfcare" OR AB “selfcare" ) OR ( TI monitor* OR AB monitor* ) OR ( TI "self management" OR AB “self management" ) OR ( TI maintenance OR AB maintenance ) OR ( TI maintain OR AB maintain ) OR ( TI behavior OR AB behavior ) OR ( TI behavior OR AB behavior ) |
| 11 | CINAHL heading | (MH "Health Behavior") OR (MH "Treatment Adherence and Compliance") OR (MH "Self-Management") OR (MH "Self Care") OR (MH "Monitoring, Physiologic") |
| 12 |  | 10 OR 11 |
| 13 |  | 9 AND 12 |
| 14 |  | TI ( myocardium OR myocardial OR ventric* OR cardial OR heart OR cardio* ) OR AB ( myocardium OR myocardial OR ventric* OR cardial OR heart OR cardio* ) |
| 15 |  | TI ( failure OR decompensat* OR insufficien* OR dysfunction* ) OR AB ( failure OR decompensat* OR insufficien* OR dysfunction* ) |
| 16 | CINAHL heading | (14 N2 15) OR (MH "Heart Failure") |
| 17 |  | 13 AND 16 |
| 18 |  | Restrict to English & published since 2008 |

**Database 3: PsycINFO (n=79)**

| **Search no.** | **Construct** | **Search terms** |
| --- | --- | --- |
| 1 | Health | ( TI health OR AB health ) OR ( TI healthcare OR AB healthcare ) |
| 2 | System | ( TI deliver* OR AB deliver* ) OR ( TI model* OR AB model* ) OR ( TI ecosystem* OR AB ecosystem* ) OR ( TI network* OR AB network* ) OR ( TI system* OR AB system* ) OR ( TI management OR AB management ) OR ( TI home OR AB home ) OR ( TI integrated OR AB integrated ) OR ( TI service* OR AB service* ) OR ( TI remote OR AB remote ) |
| 3 | APA Thesaurus of Psychological Index Terms | (DE "Health Care Delivery") OR (DE "Telemedicine") |
| 4 |  | 1 AND 2 |
| 5 |  | 3 OR 4 |
| 6 | Technology | ( TI device* OR AB device* ) OR ( TI "tele medicine" OR AB “tele medicine" OR TI telemedicine OR AB telemedicine ) OR ( TI "e health" OR AB “e health" OR TI ehealth OR AB ehealth ) OR ( TI "m health" OR AB “m health" OR TI mhealth OR AB mhealth ) OR ( TI mobile OR AB mobile ) OR ( TI "internet of things" OR AB “internet of things" ) OR ( TI "IoT" OR AB "IoT" ) OR ( TI technolog* OR AB technolog* ) OR ( TI smart OR AB smart ) OR ( TI ubiquitous OR AB ubiquitous ) OR ( TI digital OR AB digital ) |
| 7 | APA Thesaurus of Psychological Index Terms | DE "Wearable Devices" OR DE "Human Technology Interaction" OR DE "Mobile Devices" OR DE "Technology" OR DE "Digital Technology" OR DE "Internet" OR DE "Digital Interventions" OR DE "Mobile Health" |
| 8 |  | 6 OR 7 |
| 9 |  | 5 AND 8 |
| 10 |  | ( TI "self care" OR AB “self care" OR TI "selfcare" OR AB “selfcare" ) OR ( TI monitor* OR AB monitor* ) OR ( TI "self management" OR AB “self management" ) OR ( TI maintenance OR AB maintenance ) OR ( TI maintain OR AB maintain ) OR ( TI behavior OR AB behavior ) OR ( TI behavior OR AB behavior ) |
| 11 | APA Thesaurus of Psychological Index Terms | DE "Self-Care" OR DE "Health Behavior" OR DE "Self-Management" "Monitoring" OR DE "Self-Monitoring" |
| 12 |  | 10 OR 11 |
| 13 |  | 9 AND 12 |
| 14 |  | TI ( myocardium OR myocardial OR ventric* OR cardial OR heart OR cardio* ) OR AB ( myocardium OR myocardial OR ventric* OR cardial OR heart OR cardio* ) |
| 15 |  | TI ( failure OR decompensat* OR insufficien* OR dysfunction* ) OR AB ( failure OR decompensat* OR insufficien* OR dysfunction* ) |
| 16 |  | (14 N2 15) |
| 17 |  | 13 AND 16 |
| 18 |  | Restrict to English & published since 2008 |

**Database 4: Scopus (n=497)**

( TITLE-ABS-KEY ( "health" OR "healthcare" ) AND TITLE-ABS-KEY ( "delivery" OR "model" OR "ecosystem" OR "system" OR "network" OR "management" OR "home" OR "integrated" OR "service" ) AND TITLE-ABS-KEY ( "device" OR "internet of things" OR "technolog*" OR "smart" OR "digital" ) AND TITLE-ABS-KEY ( "self care" OR "monitor" OR "self management" OR "maintenance" OR "maintain" OR "behavior" OR "behavior" ) AND TITLE-ABS-KEY ( "heart failure" OR "cardiac failure" ) AND PUBYEAR > 2008 ) AND ( LIMIT-TO ( LANGUAGE , "English" ) ) AND ( EXCLUDE ( DOCTYPE , "re" ) ) = 497

**Database 5: ACM Digital Library(n=49)**

[Abstract: "heart failure"] AND [[Full Text: management] OR [Full Text: care] OR [Full Text: monitor*] OR [Full Text: maintenance] OR [Full Text: maintain*] OR [Full Text: behavior] OR [Full Text: behavior] OR [Full Text: compliance]] AND [Publication Date: (01/01/2008 TO 31/12/2021)]

**Database 6: IEEE Xplore (n=457)**

((("Document Title":"heart failure" OR heart NEAR/2 failure) OR ("Abstract":"heart failure" OR heart NEAR failure) OR ("Mesh_Terms":"Heart Failure")) AND (("Document Title": management OR care OR monitor OR maintenance OR maintain OR behavior OR compliance) OR ("Abstract": management OR care OR monitor OR maintenance OR maintain OR behavior OR compliance) OR ("Mesh_Terms":"Health Behavior" OR "Treatment Adherence and Compliance" OR "Self-Management" OR "Self-Care" OR "Monitoring, Physiologic")) AND (("Document Title":device OR health OR mobile OR technology OR "internet of things" OR "IoT" or ubiquitous OR digital OR smart OR internet) OR ("Abstract": device OR health OR mobile OR technology OR "internet of things" OR "IoT" or ubiquitous OR digital OR smart OR internet) OR ("Mesh_Terms":"Internet" OR "Internet-Based Intervention" OR "Internet of Things" OR "Digital Technology")))
