# Supplementary material for: Characteristics of Smart Health Ecosystems That Support Self-care Among People With Heart Failure: Scoping Review
Source: JMIR Cardio. 2022 Nov 2;6(2):e36773. doi: 10.2196/36773 (PMC9669885; doi:10.2196/36773)
Supplement: Multimedia Appendix 2 [file cardio_v6i2e36773_app2.docx]

#### Multimedia Appendix 2

#### Data extraction form

| **Article details** | |
| --- | --- |
| Title | Title of paper |
| Author(s) | Authors of paper |
| Year of publication | Year of publication (e.g., 2017) |
| Journal | Name of journal |
| Country | Name of country where study was conducted |
| Aims/purpose/objectives of the study | State aim of paper |
| Study type | Study design |
| **Intervention details** | |
| Name of system | State name of intervention |
| Brief description | Brief description of intervention |
| Diagnosis | Diagnosis of people intervention was designed for |
| Full description of intervention | Information about intervention reported in article |
| **Participant details** | |
| Number of participants | Number of participants |
| Age | Age of participants |
| Health status | Information about health status of participants |
| Technology use | Information about technology use/experience of participants |
| **Other** | |
| Notes | Any other information that might be relevant to answering the review questions |
