# Supplementary material for: Characteristics of Smart Health Ecosystems That Support Self-care Among People With Heart Failure: Scoping Review
Source: JMIR Cardio. 2022 Nov 2;6(2):e36773. doi: 10.2196/36773 (PMC9669885; doi:10.2196/36773)
Supplement: Multimedia Appendix 3 [file cardio_v6i2e36773_app3.pdf]

Multimedia Appendix 3

Table S3: Summary characteristics of included articles (N=34)

Intervention name	Author, year, ref no.	Study type & design	Key outcomes	Key eligibility criteria	Patient participants (n)	Intervention duration	Comparison
CONNECARE	de Batlle, 2020 [26]	Trial: Prospective, pragmatic, 2-arm, parallel, type 1 hybrid effectiveness-implementation trial	Person-centeredness<br>Continuity of care<br>Acceptability	Over 55 years with COPD or HF, passing a basic technological test and 1. history of hospitalizations, or 2. undergoing an elective hip/knee surgery	Total: 156, Control: 65, Intervention: 91<br><br>(Number with HF not specified)	3 months	Usual care (patients managed in primary care)
	de Batlle, 2021 [25]	Trial: Prospective, pragmatic, two-arm, parallel implementation trial	Change in health status<br>Healthcare use<br>Cost-effectiveness	Over 55 years with COPD or HF, passing a basic technological test	Total: 87, Control: 35, Intervention: 52<br><br>(Number with HF not specified)	3 months	Usual care (patients managed in primary care)
do Cardiac Health Advanced New Generated Ecosystem (Do CHANGE 2)	Broers, 2020 [26]	Analysis of intervention arm of RCT	Change in lifestyle and health data	Hypertension or heart failure (NYHA Class I-IV) or coronary artery disease<br><br>Exclusion: not having access to the internet or a compatible smart phone	Total included in analysis: 70 (HF:15)	6 months (210 days)	n/a
	Broers, 2020 [27]	Trial: 2-arm RCT	Feasibility of intervention (usability, acceptance, satisfaction)<br><br>Effects on lifestyle change and QoL	Aged 18-75 years with primary diagnosis of hypertension, or symptomatic heart failure, or coronary artery disease<br><br>Exclusion: not having access to the internet or a compatible smart phone	Total:150 (HF:33), Control: 74 (HF:17), Intervention: 76 (HF:16)	Total 6 months (behavioral intervention for first 3 months, technological tools for all 6 months)	Usual care (e.g. regular outpatient hospital visits)
HeartCycle Heart Failure Management (HFM) System	Maglaveras, 2011 [29]	Description of intervention design/development	-	-	-	-	-
	Reiter, 2013 [30]	Description of intervention	-	-	-	-	-

Intervention name	Author, year, ref no.	Study type & design	Key outcomes	Key eligibility criteria	Patient participants (n)	Intervention duration	Comparison
		design/developme nt					
	Reiter, 2009 [31]	Description of intervention design/developme nt	-	-	-	-	-
HeartMan	Clays, 2021 [32]	Trial: randomized controlled proof of concept trial	Effect on HRQoL, self-management, exercise capacity, illness perception, mental and sexual health	Adult ambulatory CHF patient (both ischemic or non-ischemic aetiology), stable condition, NYHA class II/III, LVEF ≤ 40%.	Total: 61, Control: 23, Intervention: 38	3 to 6 months	Usual care (standard treatment in line with clinical guidelines offered by the cardiologist, general practitioner and CHF nurse)
	Derboven, 2020 [33]	Case study analysis of intervention design process	-	-	-	-	-
	Derboven, 2018 [34]	Description of intervention development	-	-	-	-	-
	Lustrek, 2021 [35]	Description of intervention development and Trial: randomized controlled proof of concept trial	Effect on HRQoL, self-management, clinical parameters, illness perception, mental and sexual health	Adult ambulatory CHF patient (both ischemic or non-ischemic aetiology), stable condition, NYHA class II/III, LVEF ≤ 40%.	Total: 61, Control: 23, Intervention: 38	3 to 6 months	Usual care (standard treatment in line with clinical guidelines offered by the cardiologist, general practitioner and CHF nurse)
	Voorend, 2019 [36]	Qualitative interviews with sub-sample of intervention arm in RCT	-	-	-	-	-
HeartMapp	Athilingam, 2016 [38]	Description of intervention design/developme nt	-	-	-	-	-
	Athilingam, 2018 [39]	Description of intervention	-	-	-	-	-

Intervention name	Author, year, ref no.	Study type & design	Key outcomes	Key eligibility criteria	Patient participants (n)	Intervention duration	Comparison
		design/development					
	Athilingam, 2017 [40]	Trial: pilot feasibility randomized controlled trial	Patient engagement and usage, self-confidence in using intervention, usability<br>Self-care behaviour, medication adherence, HF knowledge, perception of QoL, depression	Aged over 30 years, diagnosis of CHF, NYHA II/III, recent hospitalization for CHF	Total: 18, Control: 9, Intervention: 9	4 weeks	Waitlisted control (received HF information – downloaded onto their mobile phone, encouraged to use 3 modules/week and complete 10 modules by 4 weeks. Assured that they will receive additional features at 4-week follow-up)
	Athilingam, 2016 [37]	Description of intervention development and usability assessment	-	-	-	-	-
	Di Sano, 2015 [41]	Description of intervention design/development	-	-	-	-	-
Home Automated Telemanagement (HAT) system	Finkelstein, 2012 [47]	Description of intervention design/development	-	-	-	-	-
	Finkelstein, 2011 [44]	Description of intervention design/development	-	-	-	-	-
	Finkelstein, 2010 [42]	Description of intervention design/development	-	-	-	-	-
	Finkelstein, 2011 [45]	Description of intervention design/development	-	-	-	-	-

Intervention name	Author, year, ref no.	Study type & design	Key outcomes	Key eligibility criteria	Patient participants (n)	Intervention duration	Comparison
	Finkelstein, 2010 [43]	Description of intervention design/development	-	-	-	-	-
	Finkelstein, 2010 [46]	Description of intervention design/development	-	-	-	-	-
Medly	Ware, 2018 [50]	Qualitative interviews with intervention users	-	-	-	-	-
	Ware, 2020 [48]	Pragmatic quality improvement study: pretest-posttest design	Health service utilization, clinical outcomes, QoL, self-care	Aged over 18 years, diagnosed with HF (managed by cardiologist at site clinic)	Total: 315	Up to 6 months	None
	Ware, 2019 [49]	Longitudinal mixed-methods explanatory sequential design	Patient adherence to morning readings	Enrolled in clinic program (intervention)	Total included in analysis: 231	Analysis over 1-year period	None
	Seto, 2012 [51]	Qualitative interviews with intervention users	-	-	-	-	-
	Seto, 2012 [13]	Trial: randomized controlled trial	Heart failure prognosis, self-care, QoL, hospital readmissions, nights in hospital, mortality	Over 18 years, ambulatory patients diagnosed with heart failure	Total: 100, Control: 50, Intervention: 50	6 months	Standard care (visit to clinic once every 2 weeks to once every 3-6 months)
n/a - Voice interface technology	Apergi, 2021 [52]	Pilot comparison study	Technology engagement	Over 18 years, diagnosis of HF, live in a house with WiFi	Total: 60, Intervention group 1: 30, Intervention group 2: 30	90 days	None
CardioConsult HF	de Vries, 2012 [53]	Case study	-	-	-	-	-
n/a - A home-based self-management programme	Nguyen, 2018 [54]	Description of intervention design/development	-	-	-	-	-

Intervention name	Author, year, ref no.	Study type & design	Key outcomes	Key eligibility criteria	Patient participants (n)	Intervention duration	Comparison
n/a - An eHealth self-management intervention	Sloots, 2021 [55]	Prospective pilot study	Adherence to intervention components, HRQoL, self-management behavior and knowledge, COPD self-efficacy, anxiety/depression	Over 40 years with diagnosis of both COPD and HF, able to use a tablet	Total:13	Up to 4 months	None
Veta Health	Gjeka, 2021 [56]	2 arm RCT	45-day hospital readmission	Primary or secondary diagnosis of CHF (NYHA III/IV)	Total (analytic sample): 62, Control: 15, Intervention: 47	45 days	Usual care (no detail)
n/a - An integrated, automatic home-monitoring and assist system	Klack, 2011 [57]	Description of intervention design/developme nt	-	-	-	-	-
