# Supplementary material for: Characteristics of Smart Health Ecosystems That Support Self-care Among People With Heart Failure: Scoping Review
Source: JMIR Cardio. 2022 Nov 2;6(2):e36773. doi: 10.2196/36773 (PMC9669885; doi:10.2196/36773)
Supplement: Multimedia Appendix 4 [file cardio_v6i2e36773_app4.pdf]

Multimedia Appendix 4
Table S4: Intervention characteristics (N=13)

Intervention name [primary ref]	Mode of delivery	Data collected	Tailoring /personalization	Theoretical underpinning
CONNECARE [25]	*Service user:*<br>Mobile phone (app)<br><br>*HCP:*<br>Web-based platform – accessed by portable tablet	Physiological<br>Symptom<br>Behavioral	Advice<br>Monitoring devices<br>Intervention content	Not reported
do Cardiac Health Advanced New Generated Ecosystem (Do CHANGE 2) [27]	*Service user:*<br>Mobile phone (apps, phone call. SMS)<br>CarePortal, Docobo Ltd.<br><br>*HCP:*<br>Online portal	Physiological<br>Symptom<br>Behavioral<br>Other *(GPS, personality)*	Advice<br>Timing of delivery<br>Mode of delivery<br>Monitoring devices	Do Something Different (DSD) behavior change program
HeartCycle Heart Failure Management (HFM) System [30]	*Service user:*<br>Device connected to TV (Philips Motiva)<br><br>*HCP:*<br>Web-based platform	Physiological<br>Symptom<br>Other *(comprehension and motivation)*	Advice<br>Content	Self-regulation theory
HeartMan [35]	*Service user:*<br>Mobile phone (app, phone call)<br>Wristband display (custom wristband)<br>Pill organizer (PuTwo, 7-Day AM/PM Night Reminder Medi-Planner)<br><br>*HCP:*<br>Web application	Physiological<br>Behavioral<br>Other *(temperature, humidity, voice recording)*	Advice<br>Timing of delivery<br>Intervention content	Cognitive-behavioral therapy, theory of cognitive dissonance

Intervention name [primary ref]	Mode of delivery	Data collected	Tailoring /personalization	Theoretical underpinning
HeartMapp [40]	*Service user:*<br>Mobile phone (app)<br><br>*HCP:*<br>Not reported	Physiological<br>Symptom<br>Behavioral	Advice<br>Intervention content	Carmen's multidimensional framework of patient engagement, intervention-motivation-behavior (IMB) model<br><br>Successive Approximation Model, Mayer's Cognitive Theory of Multimedia Learning, Sweller's Cognitive Load Theory, Instructional Design Approach utilizing a Pedagogical Agent, Problem based learning
Home Automated Telemanagement (HAT) system [42]	*Service user:*<br>Home unit (notebook computer, PlayStation, Xbox, or Wii)<br><br>*HCP:*<br>Clinician unit<br>Email	Physiological<br>Symptom<br>Behavioral	Advice<br>Alerts	Model of chronic disease care
Medly [48]	*Service user:*<br>Mobile phone (app, automated phone call)<br><br>*HCP:*<br>Web dashboard<br>Email	Physiological<br>Symptom	Advice<br>Alerts	Connelly Framework for Self-Care in Chronic Illness
N/A[a] - Voice interface technology [52]	*Service user:*<br>Conversational agent (Alexa)<br><br>*HCP:*<br>Email and text (alerts)	Symptom<br>Behavioral	Advice<br>Timing of delivery	Not reported
CardioConsult HF [53]	*Service user:*<br>Health monitor (Turnstall)<br><br>*HCP:*<br>SMS<br>Email<br>DSM system (computer)	Physiological<br>Symptom	Advice	Not reported

Intervention name [primary ref]	Mode of delivery	Data collected	Tailoring /personalization	Theoretical underpinning
N/A - A home-based self-management programme [54]	*Service user:*<br>Mobile phone (app)<br><br>*HCP:*<br>Not reported	Physiological<br>Behavioral	Advice<br>Intervention content	Activity theory
N/A - An eHealth self-management intervention [55]	*Service user:*<br>Tablet (app)<br>Respiro, Amiko Digital Health add-on inhaler sensor<br>Face to face (individual and group training sessions)<br>Phone call<br><br>*HCP:*<br>Website	Physiological<br>Symptom<br>Behavioral<br>Other *(depression & anxiety)*	Advice<br>Monitoring devices	Not reported
Veta Health [56]	*Service user:*<br>Mobile phone (app)<br><br>*HCP:*<br>Veta Health platform (computer)	Physiological<br>Symptom<br>Behavioral	Advice	Not reported
N/A - An integrated, automatic home-monitoring and assist system [57]	*Service user:*<br>Interactive wall display<br>Video call<br><br>*HCP:*<br>Not reported	Physiological<br>Behavioral	Advice<br>Alerts	Not reported

[a]N/A: not applicable.
